# Breast cancer and breast cancer screening use—beliefs and behaviours in a nationwide study in Malaysia

**DOI:** 10.1186/s12889-023-16227-0

**Published:** 2023-07-10

**Authors:** Min Min Tan, Aminatul Saadiah Abdul Jamil, Roshidi Ismail, Michael Donnelly, Tin Tin Su

**Affiliations:** 1 South East Asia Community Observatory (SEACO), Jeffrey Cheah School of Medicine and Health Sciences, Monash University Malaysia, Jalan Lagoon Selatan, Bandar Sunway, 47500 Selangor, Malaysia; 2 Health Industry Technology Programme, Faculty of Science and Technology, Universiti Sains Islam Malaysia, Negeri Sembilan, Malaysia; 3 Centre for Population Health (CePH), Department of Social and Preventive Medicine, Faculty of Medicine, University of Malaya, Kuala Lumpur, Malaysia; 4 Centre for Public Health, Queen’s University Belfast, Northern Ireland, UK

**Keywords:** Breast cancer screening, Beliefs, Cancer prevention

## Abstract

**Background:**

Many upper-middle-income countries (UMICs), including Malaysia, continue to face low breast cancer (BC) screening rates and patients with delayed presentation of BC. This study investigated the role of beliefs about BC and use of screening (e.g. beliefs about whether or not screening reduced the possibility of dying from BC).

**Methods:**

A nationwide cross-sectional study was conducted in which a total of 813 women (aged ≥ 40 years old) were randomly selected and surveyed using the validated Awareness and Beliefs about Cancer (ABC) measure. The association between BC screening use, sociodemographic characteristics, and negative beliefs about BC screening were analysed using stepwise Poisson regressions.

**Results:**

Seven out of ten Malaysian women believed that BC screening was necessary only when experiencing cancer symptoms. Women > 50 years and from households with more than one car or motorcycle were 1.6 times more likely to attend a mammogram or a clinical breast examination (mammogram: Prevalence Ratio (PR) = 1.60, 95% Confidence Interval (CI) = 1.19–2.14, Clinical Breast Examination (CBE): PR = 1.61, 95% CI = 1.29–1.99). About 23% of women expected to feel anxious about attending BC screening, leading them to avoid the procedure. Women who held negative beliefs about BC screening were 37% less likely to attend a mammogram (PR = 0.63, 95% CI = 0.42–0.94) and 24% less likely to seek a CBE (PR = 0.75, 95% CI = 0.60–0.95).

**Conclusions:**

Public health strategies or behaviour interventions targeting negative beliefs about BC screening among Malaysian women may increase uptake and reduce late presentation and advanced-stage cancer. Insights from the study suggest that women under 50 years, in the lower income group without a car or motorcycle ownership, and of Malay or Indian ethnicity (compared to Chinese-Malay) are more likely to hold beliefs inhibiting BC screening.

## Introduction

Globally, cancer is a leading cause of death – accounting for about 10 million deaths in 2020 [1]. Cancer mortality is predicted to increase to 17 million by 2030 due to population ageing and unhealthy lifestyles [2]. According to the GLOBOCAN 2020 report, breast cancer (BC) is the most prevalent type of cancer in Malaysian women (32.9 per cent), followed by colorectal cancer (11.9 per cent) and ovarian cancer (7.2 per cent) [3]. Malaysian women also tend to present late with breast cancer; more than half (52.2%) of the female population present with advanced-stage cancer [4, 5]. Delays between symptom onset and treatment lead to poorer BC survival [6, 7]. BC screening increases the chance that malignant tumours will be detected and treated earlier to improve survival. For example, a study of 2,796,472 Canadian women aged 35 to 44 years found that mammogram screenings were associated with substantially reduced breast cancer mortality [7]. In Finland, females aged 40–74 who attended mammogram screenings also had a significantly higher survival rate than women who did not screen [8].

Beliefs about health and illness have the potential to affect health behaviours, for example, visiting a primary care physician and availing of screening [9]. Models and theories about behavior such as the Health Belief Model and related research point to the role of beliefs and cognitions in terms of contributing to explanations for healthy or risk-taking behaviours including whether or not to follow a physician’s recommendation or offer of BC screening. More specifically, negative beliefs about BC may contribute to a longer patient interval between recognising symptoms and visiting a physician or healthcare practitioner [10]. Conversely, positive beliefs about the benefit of early cancer detection may increase the likelihood of healthcare-seeking behaviours including screening uptake [11, 12]. Identifying and understanding negative beliefs or misbeliefs about BC and positive beliefs, for example, the belief in early BC detection benefits will provide evidence to inform public health strategies designed to increase screening behaviour, reduce the patient interval, lead to earlier presentation and detection, and improve patient treatment experience and outcomes and higher survival rates [13, 14]. Whilst the Ministry of Health funds the provision and delivery of opportunistic BC screening, BC remains the most common cancer in Malaysia. There is a paucity of studies about beliefs regarding BC screening in Malaysia and, generally, in Southeast Asia. Thus, the purpose of the study that is presented in this paper was to investigate the role of beliefs in BC screening behaviour.

## Methods and materials

### Sampling

The current study was part of a larger research programme designed to ‘benchmark’ knowledge about cancer signs/symptoms and risk factors among the Malaysian population using the *Awareness and Beliefs about Cancer (ABC) measure* which was validated and culturally adapted for use in Malaysia [15, 16]. The random digit dialing (RDD) technique for telephone studies [17] was used to construct a sampling frame that captured all members of the Malaysian population with a mobile phone number based on operator prefixes. Next, we selected a probability sample of 150,000 people with phone numbers. We randomly generated 150,000 phone numbers and screened them for activated Subscriber Identity Module (SIM) cards, resulting in a sample of 55,000 active mobile phone numbers. From this sample, 10,000 numbers were selected for initial calls. Using Computer Assisted Telephone Interviewing (CATI) software from Creative Research System [18], trained interviewers contacted 8,406 active mobile numbers. Out of the attempted calls, 4,913 were answered, and among those, 3,254 eligible individuals (58%) agreed to participate in the survey. Among the respondents, there were 813 women, accounting for 43% of the overall sample and included in the analysis of this study. Further details about the sampling and data collection methods have been described in previous studies [16, 19].

The ABC Malaysia questionnaire was culturally adapted for utilization in Malaysia, including contextual translation into Malay, Mandarin, and Tamil languages. Additionally, cognitive interviewing was conducted to ensure respondents’ comprehension. For this study, a team of six staff members were trained as interviewers, comprising two native speakers of Malay, three native speakers of Mandarin and Cantonese, and one native speaker of Tamil. All six interviewers are also proficient in English. All participants provided verbal, voluntary informed consent to participate in a telephone interview during which they were free to stop at any time. Ethical approval was granted by the University of Malaya Medical Centre Ethics Committee (Reference Number 890.6).

Data were weighted and stratified according to sociodemographic characteristics such as age, sex and ethnic group to ensure that the sample of respondents was representative of the Malaysian population according to the national census in 2010 [20]. Regarding CBE uptake, based on BC screening participation in the past five years [21], it was estimated that a sample size of 1000 women would be required detect a 10 per cent difference with 80 per cent power between people with positive and negative beliefs. This calculation assumed that 55 per cent of women with positive beliefs and 30 per cent with negative beliefs would take up the offer of screening. Regarding mammogram uptake, it was estimated that a sample of 1000 women would be required to detect an 8 per cent difference with 80 per cent power between those with positive and negative beliefs—based on the assumption of an uptake proportion of 23 per cent of women with positive beliefs and 30 per cent with negative beliefs. The final sample collected of study was n = 813 participants who are female aged 40 years old and above. This age group was chosen as cancer incidence is higher in people aged 40 years and above in Malaysia. A power analysis was performed prior to the study to determine the required sample size for detecting a statistically significant effect. The final sample size collected (*n* = 813) fell slightly short of the estimated size of 1000 women. Nevertheless, the slightly smaller sample of 813 women is expected to be adequate in detecting the anticipated effect of positive and negative beliefs in BC screening, considering the conservative estimates used in the initial power calculation.

### Data collection

The average time taken for the interview was 20–30 min. The significant barriers or challenges that the research team faced doing this study include the length of time in convincing the participants to agree to the study and set up a follow-up appointment, the low participation rate of phone interviews and technical issues encountered during the phone calls. The research involves the use of coded personal information and phone numbers, however, the confidentiality of the participants was upheld by not disclosing any personal information without the consent or knowledge of the individuals. The collected phone numbers and data were stored in accordance with University of Malaya regulations. All the personal data were anonymized and stored on a server that is further encrypted. Only the Database Manager (RI) has access to the complete data set. Access to the phone numbers in the CATI software was secured with a password and the numbers were deleted permanently after conducting a security check. The phone numbers generated by the Random Digit Dialing in the CATI software were not stored long term and were deleted immediately after the conclusion of the study. The dataset for the study were retained for 5 years before being destroyed. The privacy of the participants was protected by storing and sharing the data only with the research team members. The raw data was not appropriate to share in a data repository, registry or open-source platform. However, the data can be shared based on reasonable request after removing identifiable information to adhere to the strict confidentiality of the participants’ information. Digital data in the CATI software was destroyed by deleting or overwriting information and ensuring the destruction of data is irreversible with no chance of recovery later. Paper copies of the phone numbers and records were shredded using secure shredding to prevent any misuse of the information, and in all cases, records relating to what was destroyed, when, and how were retained.

### Measures

#### Cancer screening uptake and beliefs about BC

A culturally validated 9-item subscale of the *Awareness and Beliefs about Cancer* measure, Malaysian version (ABC-M) [14, 15] was used to assess uptake of, and awareness and beliefs about, BC screening. For instance, the ABC-M asked women if in the past five years they had attended (i) BC screening (yes/no) or received a mammogram (yes/no); and if they had had a CBE in the past year (yes/no). Three items measured beliefs about BC screening: 1) were women worried about what might be found at BC screening; 2) was BC screening necessary only if and when a woman had symptoms, and 3) did uptake of BC screening reduce the chance of dying from BC? Each item was scored on a 4-point scale from strongly disagree to strongly agree. Responses at each end of the Likert scale were endorsed infrequently and, therefore, the items were dichotomised into a binary variable (disagree and agree). Finally, the participants were asked about their age, gender, ethnicity, education, marital status, smoking status, household ownership of a motorised vehicle and whether or not they had received a BC diagnosis or if a family member or friend had or had had BC.

### Analyses

Descriptive statistics were reported with weighted means and percentages. Multiple imputations by chained equations were conducted to replace missing values [22]. Then, stepwise Poisson regressions were conducted to examine the association between BC screening attendance, sociodemographic characteristics and beliefs about BC screening. Associations between the outcome and the explanatory variables were explored first in univariate analyses. Subsequently, in multivariate Model 1, only variables with p values of < 0.2 in the univariate analyses were included; and then, in multivariate Model 2, only variables with p values of < 0.2 in Model 1 were included. The effect measures were weighted and reported with 95% confidence intervals and their standard errors were calculated as robust variance. All analyses were carried out using STATA v13.

## Results

### Descriptive findings

Out of the eligible individuals, 3,254 (58%) agreed to participate in the survey. As for the response rate, 813 women completed the survey at the end of the study and were included in the analysis, making up 43% of the sample. Table 1 provides a summary of the characteristics of 813 females who completed the ABC survey. After post-stratification, about 50% of respondents were Malays and 45% were between 40–49 years old. Approximately 29% of respondents attended tertiary education, and most women were married (92.1%). More than half of respondents owned one motor vehicle (51.9%). One fifth (21%) of respondents had an experience of cancer (including indirectly via immediate family, relatives, or friends).

**Table 1:** Characteristics of participants (*n* = 813)

	**Number**	**Percentage**	**Weighted Percentage**^a^
**Age groups** (years)			
40–49	573	70.5	45.0
50–59	178	21.9	32.1
> 60	62	7.6	22.9
**Ethnicity**			
Malay	391	48.1	51.4
Chinese	341	42.0	33.3
Indian	62	7.6	6.9
Others	19	2.3	8.3
**Highest level of education**			
No formal education	18	2.2	5.2
Primary education	74	9.1	15
Secondary education	423	52	49.6
Tertiary education	298	36.7	30.2
**Marital status**			
Married	728	89.5	92.1
Not married	85	10.5	7.9
**Ever smoked**			
Yes	801	98.5	97.6
No	12	1.5	2.4
**Household ownership of motor vehicle**			
No	38	4.7	7.3
Yes, one	448	55.1	51.9
Yes, more than one	327	40.2	40.8
**Experience of cancer** (self, family or friend)			
Yes	181	22.3	24.3
No	632	77.7	75.7

^a^Post-stratification weight using population age, sex and ethnicity distribution of  > 40 years old Malaysian from the 2010 census report

Only 29% (95% CI, 24.7%-33.2%) of women aged 40 and above and 35% (95% CI, 27.6–41.5%) above 50 years old underwent a mammogram in the past 5 years, and 39.8% (95% CI, 35.4%-44.2%) of women had a CBE in the previous year (Table 2). No significant differences were found (based on sex, ethnicity, education, and marital status) between respondents who attended and did not attend BC screening. A significantly higher percentage of women at 40 years and above who had had experience with cancer received a mammogram (37.2% vs 26.6%) and a CBE (50.3% vs 36.7%) compared to respondents without previous cancer experience (Table 2).

**Table 2:** Frequency and weighted percentage of mammogram and CBE uptake by personal cancer experience

	**Personal experience with cancer**				**Total**	
	**Yes**		**No**			
**Type of screening**	***n***	**Weighted % (95%CI)**	***n***	**Weighted % (95%CI)**	***n***	**Weighted % (95%CI)**
Attended mammogram in the past 5 years (women ≥ 40 years old)	58/181	37.2 (28.6–46.7)	142/629	26.6 (22.1–31.6)	200/810	29.2 (25.1–33.6)
Attended mammogram in the past 5 years (women ≥ 50 years old)	31/71	43.4 (31.1–56.6)	52/115	31.6 (24.1–40.3)	83/238	35.0 (28.4–42.2)
Had a clinical breast examination in the past year	96/181	50.3 (41.3–59.4)	221/630	36.7 (31.9–41.8)	317/811	40.0 (35.7–44.5)

A significantly higher percentage of women who experienced cancer were worried about what might be found during a mammogram than women without any cancer experience (32.2% vs 18.4%). A significantly lower percentage of women who experienced cancer agreed that BC screening was necessary only when symptoms appeared (64.4% vs 78.6%). A slightly lower percentage of women who experienced BC agreed that having a mammogram or CBE screening could reduce the possibility of dying from BC (80.3% vs 85.2%).

Regarding negative beliefs about BC screening, about 23.2% of women (95% CI, 19.5%-27.3%) were ‘worried so much about what might be found at a mammogram and a CBE that they preferred not to have it’ (Table 3); 70.7% (95% CI, 66.2%-74.8%) believed that mammograms were necessary only if there were BC symptoms. There was disbelief among 14.8% (95% CI, 11.7%-18.5%) of women that mammograms could reduce the chance of dying from breast cancer.

**Table 3:** Frequency and weighted percentage of negative beliefs about mammogram and CBE screening by personal cancer experience

	**Personal experience with cancer**				**Total**	
**Beliefs about BC screening**	**Yes**		**No**			
	***n***	**Weighted % (95% CI)**	***n***	**Weighted % (95% CI)**	***n***	**Weighted % (95% CI)**
I am so worried about what might be found at a mammogram and CBE that I prefer not to have it. (agree)	58/180	30.8 (23.2–39.7)	117/627	20.8 (16.7–25.5)	175/807	23.2 (19.5–27.3)
Mammogram or CBE is necessary only if I have symptoms (agree)	116/180	62.2 (53.1–70.5)	494/628	73.5 (68.2–78.1)	610/808	70.7 (66.2–74.8)
A mammogram or a CBE could reduce my chance of dying from breast cancer (disagree)	35/178	20.5 (14.0–29.0)	73/620	13.0 (9.7–17.2)	108/798	14.8 (11.7–18.5)

### Association between negative beliefs about BC screening and mammogram attendance

Age group, being married, household ownership of a motor vehicle and past cancer experience were significantly associated with mammogram attendance in the past five years (Table 4). Women who were so worried about what might be found at BC screening that they preferred to forgo it and who believed that BC screening was necessary only after the self-discovery of symptoms were significantly less likely to have attended a mammogram previously. Positive beliefs about BC screening reducing the chance of dying from breast cancer were significantly associated with mammogram attendance.

**Table 4:** The association between beliefs about breast screening, demographic variables and mammogram attendance

**Variable**	**PR (95% CI)**^a^	**PR (95% CI)**^b^	**PR (95% CI)**^c^
**Age group**			
40–49	1.00		
50 and above	1.56 (1.19–2.04)**	1.54 (1.17–2.03)**	1.59 (1.22–2.06)**
**Ethnicity**			
Malay	1.00		
Chinese	0.91 (0.67–1.24)		
Indian	1.14 (0.72–1.80)		
Other	1.57 (0.88–2.81)		
**University education**			
No	1.00		
Yes	1.24 (0.88–1.76)*	1.41 (0.78–2.55)	
**Marital status**			
No	1.00	1.00	
Yes	1.65 (0.87–3.13)*	1.41 (0.78–2.55)	
**Household ownership of motor transport**			
One or none	1.00	1.00	1.00
More than one	1.60 (1.19–2.14)**	1.53 (1.13–2.06)**	1.59 (1.19–2.11)**
**Smoking status**			
Ever smoked	1.00		
Never smoked	2.24 (0.55–9.13)		
**Experience of cancer** (self, family or friend)			
No	1.00	1.00	
Yes	1.41 (1.04–1.92)*	1.33 (0.97–1.82)*	
**Beliefs about BC screening**			
Worried what might be found			
Disagreed	1.00	1.00	1.00
Agreed	0.63 (0.42–0.94)**	0.63 (0.42–0.93)**	0.63 (0.42–0.94)**
Necessary only if I have symptoms			
Disagreed	1.00		
Agreed	0.89 (0.65–1.22)		
Reduces chance of dying from breast cancer			
Disagreed	1.00	1.00	
Agreed	0.80 (0.54–1.17)*	0.82 (0.55–1.23)	

^*^p < 0.2, **p < 0.05

^a^Univariate analysis

^b^Multivariate Model 1, variables with *p* < 0.2 in univariate analyses were included

^c^Multivariate Model 2, variables with *p* < 0.2 in multivariate Model 1 were included

Regarding the results of the multivariate analyses (Model 1 and 2), women aged 50 and above and from households owning more than one motorised transport were 1.6 times more likely to have attended mammogram screening (PR = 1.60, 95% CI = 1.19–2.14, 95% CI = 1.19–2.11) (Table 4). The belief item relating to worry about what might be found at screening was statistically significant – women who endorse this item were less likely to have attended a mammogram (PR = 0.63, 95% CI = 0.42–0.94). Also statistically significant were the results for the other two belief items, that BC screening is only necessary if they have symptoms (PR = 0.89, 95% CI = 0.65–1.22) and that BC screening reduces the chance of dying from breast cancer (PR = 0.80, 95% CI = 0.54–1.17, 95% CI = 0.55–1.23).

There were significant differences between ethnic groups in terms of holding negative beliefs about BC screening. More Chinese (5.7%) compared to Malays (0.5%) and Indians (0%) disagreed with each one of the three belief items.

### Association between beliefs about BC screening and CBE

Table 5 indicates that women from households who owned more than one motor transport were 1.6 times more likely to attend a CBE (PR = 1.61, 95% CI 1.29–1.99). Previous experience with cancer was significantly associated with CBE attendance (PR = 1.38, 95% CI 1.10–1.73) but women who believed that BC screening was necessary only if symptoms were present were significantly less likely to have attended CBE. The multivariate Model 1 analysis confirmed these results—women with motor transport (PR = 1.58, 95% CI = 1.26–1.99) and previous cancer experience (PR = 1.27, 95% CI = 1.00–1.60) were more likely to have attended a CBE though beliefs about BC screening were not significantly associated with CBE attendance.

**Table 5:** The association between clinical breast examination (CBE), demographic variables, and negative beliefs about BC screening

**Variable**	**PR (95% CI)**^a^	**PR (95% CI)**^b^	**PR (95% CI)**^c^
**Age group**			
40–49	1.00		
50 and above	1.13 (0.92–1.39)*	1.07 (0.87–1.32)	
**Ethnicity**			
Malay	1.00		
Chinese	0.87 (0.69–1.09)		
Indian	0.91 (0.62–1.35)		
Other	1.19 (0.71–1.99)		
**University education**			
No	1.00	1.00	
Yes	1.24 (0.96–1.59)*	1.19 (0.90–1.57)	
**Marital status**			
No	1.00	1.00	
Yes	1.25 (0.86–1.81)*	1.28 (0.89–1.86)	
**Household ownership of motor transport**			
One or none	1.00	1.00	1.00
More than one	1.61 (0.130–2.00)**	1.58 (1.26–1.99)**	1.61 (1.29–1.99)**
**Smoking status**			
Never smoked	1.00		
Ever smoked	0.98 (0.41–2.33)		
**Experience of cancer** (self, family or friend)			
No	1.00	1.00	1.00
Yes	1.38 (1.10–1.73)**	1.27 (1.00–1.60)**	1.29 (1.02–1.63)**
**Beliefs about BC screening**			
Worried what might be found			
Disagreed	1.00		
Agreed	1.14 (0.88–1.47)		
Necessary only if I have symptoms			
Disagreed	1.00		
Agreed	0.75 (0.60–0.95)**	0.80 (0.73–1.03)*	0.76 (0.60–0.95)**
Reduces chances of dying from breast cancer			
Disagreed	1.00	1.00	
Agreed	0.73 (0.56–0.96)**	0.80 (0.59–1.08)	

^*^p < 0.2, **p < 0.05

^a^Univariate analysis

^b^Multivariate Model 1, variables with *p* < 0.2 in univariate analyses were included

^c^Multivariate Model 2, variables with *p* < 0.2 in multivariate Model 1 were included

## Discussion

The study uncovered concerning findings regarding BC screening. Seven out of ten women believed that BC screening was necessary only when symptoms were present, which is alarming considering the high proportion of women presenting with advanced stage BC in Malaysia. However, this finding provides an opportunity to address late presentation byimplementing interventions aimed at dispelling misbeliefs, raising awareness, increasing knowledge and improving BC screening uptake. The results are consistent with studies conducted in Asia, such as a community-based intervention study in South Korea where even higher proportions of women held similar beliefs that a mammogram was unnecessary when they were asymptomatic [23].

The participants seemed unaware about how their beliefs could restrict help-seeking behaviour and negatively impact their health. While 85% of the respondents agreed that BC screening could reduce the risk of death, they did not understand the purpose of screening or to connect their beliefs about reduced risk due with the importance of using screening services when asymptomatic. The response pattern indicated a level of uncertainty among Malaysian women regarding BC and BC screening uptake. Previous studies in Malaysia and Singapore have shown a high prevalence of fatalistic beliefs related to cancer [24–26], which may act as a barrier to early cancer detection and treatment. Chinese women in particular held more negative beliefs about BC screening items (5.7% of Chinese, 0.5% of Malays, and 0% of Indians), due perhaps to cultural differences in cancer fatalism.

Women who believed that BC screening was necessary only when symptoms appeared were less likely to have received a CBE in the past five years.Generally, women are more aware of cancer than men, but may face more barriers to cancer presentation and screening uptake, including emotional barriers [27, 28]. Women compared to men also are more likely to hold negative cancer beliefs [10] and perceived barriers have been shown to affect mammogram utilization [29].

It is concerning that a significant proportion of women in the study (24%) reported a preference to forgo screening due to worry about the outcome. This worry acts as a barrier to BC screening uptake, despite the significant risk reduction of dying from breast cancer within 10 years associated with participation in mammogram screening [30]. The participants in our study who held this belief or worried in this way were 40% less likely to have attended mammogram screenings in the past five years. Fear of the outcome of screening tests has been shown to hinder mammogram screening in other studies [31–33] though some studies found that fear may serve both as a facilitator and an obstacle to help-seeking [34, 35]. The Yerkes–Dodson law may offer an explanation for these findings in terms of the level of fear intensity or anxious feelings experienced by women – a moderate level of anxiety may act as a motivator whilst too much fear and anxiety may restrict or inhibit help-seeking behaviour [36, 37]. A study in Canada found a moderate level of worry positively influenced mammogram and CBE uptake among women at familial risk of BC [38].

Help-seeking behaviour such as attending BC screening appears more likely when women experience a certain level of anxiety and also feel self-efficacious in dealing with a cancer diagnosis [39]. This study suggest that previous experience with breast cancer may contribute to increased worry about screening test results, but it also appears to improve understanding about the role of screening in early cancer detection.. The ABC measure did not enquire about the nature of previous BC experiences though it is reasonable to suggest that previous experience may heighten perceptions about an increased risk of BC and related worries and apprehensions. Indeed, research supports the view that having a family or friend with BC increases personal perceived risk [40], responsiveness to cues to action for screening [41] and adherence to cancer screening [42]. Involving cancer survivors and relatives in public health messages about the value of screening can potentially increase uptake among Malaysian women.

While population-based BC screening has contributed to reduced cancer mortality in high-income countries [42, 43],the delivery of BC screening in Malaysia and most Asian low- and middle-income countries is opportunistic, resulting in poor uptake, delayed presentation, treatment delays and poorer survival rates [44]. The self-reported mammogram attendance in this study (29%) was higher compared to previous Malaysian studies (7% to 25%) [35] and comparable to other countries that do not offer population-based screening (12–31% in Brazil, 35% in a region of Switzerland) [45, 46]. Older women (≥ 50 years old) were significantly more likely to attend mammogram screening [47]. Compared to countries with population-based screening, a significantly lower proportion of Malaysians engaged in screening (66% in Germany, 75% in Spain, 78% in a region of Switzerland) [46, 48, 49], indicating low awareness of BC symptoms other than breast lumps [50, 51].

BC screening services in Malaysia are available for a nominal fee at public healthcare facilities, which may explain the higher uptake compared to colorectal cancer screening (29% vs 28%) [52]. BC awareness campaigns in Malaysia have had wider dissemination, longer duration and higher frequency [50], but low BC screening use and delayed presentation of BC persist. Further research is needed to clarify the role of primary care physicians in screening adherence [53].

This study did not show an association between being university-educated and BC screening uptake, unlike Western countries [54], potentially due to cultural differences and fatalism-related beliefs [55]. Access issues also influence screening uptake, with ownership of multiple motor vehicles associated with higher use of BC screening. Longer travelling distance to BC services is linked to lower likelihood of screening [35], even when it is offered free of charge [56] and performed at a more advanced stage of BC [57].

The study has limitations that should be acknowledged. The recruitment process resulted in a low enrolment rate of 43% (*n* = 813/1895) due to attrition during follow-up interviews. The low response rate may be attributed to reliance on telephone surveys, which presented technical barriers such as dropped calls and unreliable telecommunication lines. Face-to-face data collection may have yielded a higher response rate, particularly for sensitive topics like breast cancer, as it may have facilitated the establishment of trust and rapport with the participants, encouraging greater openness in sharing beliefs and experiences.

Bearing in mind the above barrier and limitations, the present study nevertheless has important public health implications, highlighting the potential for BC screening to improve cancer survival rates [7, 8], the need to address beliefs about BC screening and raise awareness of the preventative value of early screening, particularly for women of 40 years old and above. Thus, tailored interventions should target misbeliefs, reduce anxiety about mammogram screening and BC screening tests results, and consider different screening modalities. The researchers recommend future research focusing on interventions targeting misbeliefs in BC screening, the relationship between beliefs about reduced BC risk and BC screening uptake, and the appropriateness of offering BC screening services to Malaysian women who are asymptomatic. BC screening programs should aim to increase self-efficacy, promote preventive action, and address access issues in suburban and rural areas to improve BC screening rates and overall population health in Malaysia. In conclusion, negative beliefs about BC screening contribute to the low uptake of BC screening behaviours among Malaysian women, as in other low and middle-income countries.

## Data Availability

Data are available on request from the corresponding authors, M Donnelly or TT Su, upon reasonable request.
